# Recommendations for future research exploring e‐cigarette use and later cigarette smoking in young people: Results from a consultation exercise

**DOI:** 10.1111/add.70038

**Published:** 2025-03-05

**Authors:** Monserrat Conde, Michael F. Pesko, Lion Shahab, Rachna Begh, Nicola Lindson, Sarah E. Jackson, Dimitra Kale, Dylan Kneale, Jonathan Livingstone‐Banks, Jamie Hartmann‐Boyce

**Affiliations:** ^1^ Nuffield Department of Primary Care Health Sciences University of Oxford Oxford UK; ^2^ Department of Economics University of Missouri Columbia MO USA; ^3^ Department of Behavioural Science and Health University College London London UK; ^4^ Evidence for Policy and Practice Information Centre (EPPI‐Centre), Social Science Research Unit, UCL Institute of Education University College London UK; ^5^ Department of Health Promotion and Policy University of Massachusetts Amherst Amherst MA USA

**Keywords:** electronic cigarettes, recommendations, smoking, vaping, young people, youth

## Abstract

**Background and Aims:**

Exploring the relationship between vaping and smoking in young people is a key area of research that can inform e‐cigarette policy. Rigorous evidence mapping and synthesis have highlighted gaps and methodological concerns in the evidence base. This study provides recommendations for the conduct and reporting of future quantitative primary research exploring e‐cigarette use and later cigarette smoking in young people (≤29 years).

**Methods:**

We developed a draft version of recommendations based on the critical appraisal of studies, findings of a systematic review and an evidence and gap map. We used an anonymized on‐line survey to run a consultation exercise with stakeholders, including researchers, non‐profit/charity workers and clinicians. Respondents rated the perceived importance of each draft recommendation on a 5‐point Likert scale and provided open‐ended comments, where relevant. We developed a final set of recommendations based on this stakeholder input.

**Results:**

We initially came up with a list of 22 recommendations, which 36 stakeholders rated in the on‐line survey. Most were researchers (*n* = 26) and from the USA (*n* = 18). Following feedback, this resulted in a final set of 23 recommendations, including recommendations for planning, data collection, data analysis and reporting. Examining causes of differences in vaping‐smoking associations, including equity factors (e.g. socioeconomic status) and contextual factors (e.g. jurisdiction levels), and generating representative longitudinal data from countries other than the USA, Canada and UK, particularly low‐ and middle‐income countries, were strongly endorsed recommendations. A new recommendation to report characteristics of e‐cigarettes (e.g. flavours) was added.

**Conclusions:**

This study provides 23 recommendations for conducting and reporting future quantitative research exploring e‐cigarette use/availability and later combustible cigarette smoking in young people. Most of the recommendations are specific to studies using repeat cross‐sectional data tracking population trends and to longitudinal cohort studies tracking behaviours in individuals.

## INTRODUCTION

In recent years, the use of e‐cigarettes has increased considerably worldwide, particularly among young people [1]. However, tobacco use – and combustible tobacco smoking in particular – is in decline in many high‐income countries [2]. There is considerable uncertainty surrounding the connection between, and magnitude of the impact of, e‐cigarettes and the use of combustible tobacco products, with most concerns being centred upon youth [3]. Some systematic reviews have reported a positive association between e‐cigarette use and later cigarette smoking in young people [4, 5], with natural experiments displaying inverse findings [6]. In addition, the lack of long‐term data on the comparative risks of e‐cigarettes versus combustible tobacco underscores the importance of this area in tobacco control. High‐quality evidence to inform policy is critical. Our evidence and gap map, and systematic review exploring e‐cigarette use/availability and later cigarette smoking in young people, have underlined areas for methodological and reporting improvement in the current evidence base [7, 8]. Developing recommendations, based on this previous work, is a rational progression to enhance the standardization, transparency and quality of the evidence base in this area. Therefore, our aim was to produce a set of recommendations for the conduct and reporting of future quantitative primary research exploring links between e‐cigarette use and later cigarette smoking in people under 30 years of age.

## METHODS

The protocol for this work was pre‐registered on the Open Science Framework (https://osf.io/rjxmp). Ethics review was considered unnecessary by the University of Oxford Medical Sciences Division Ethics committee. However, principles of ethical research were followed for the stakeholder engagement exercise.

The author team drafted initial recommendations based on the findings of a systematic review [8] and an evidence and gap map (EGM) [7] on the impact of any type of e‐cigarette use or availability of e‐cigarettes on subsequent cigarette smoking in young people. Our literature search was up to date to 3 April 2023. We critically appraised the quality of included studies using a fit for purpose risk‐of‐bias (RoB) tool adapted from the RoB instrument published by Morgan *et al*. in 2019 [9]. All 126 studies included in our review were judged to be at moderate or higher RoB, with the RoB criteria informing our draft recommendations. EGMs are interactive tools that allow the visualization of clusters and gaps of evidence in a specific area of research [7]. We adapted the Campbell Collaboration and International Initiative for Impact Evaluation (3ie) methods [7]. The conceptual framework and domains of the EGM were informed by consultation exercises involving stakeholders in the field of tobacco control and policy (policy officers, representatives of Cancer Research UK (CRUK), practitioners and researchers). The gaps identified included a lack of studies outside the USA, Canada and the UK – particularly in low‐ or middle‐income countries (LMICs) – and a lack of analyses regarding sociodemographic and contextual factors.

One researcher (M.C.) initially analysed the items that had consistently scored a higher risk of bias in the included studies of the systematic review, the findings and recommendations for future research of the systematic review [8], and the relative and absolute gaps identified in the EGM [7]. The first list of draft recommendations was compiled by grouping and cross‐checking this information across the three supporting materials. It was then revised and discussed with the principal investigator (J.H.B.). The resulting document was subsequently circulated to the rest of the author team for feedback and subsequent changes, until a final draft version was reached through interactive discussion. We proposed 22 original recommendations (for the recommendations and supporting sources, see Table 1).

**TABLE 1:** Recommendations for studies using repeat cross‐sectional data tracking population trends.

Final recommendation	Original recommendation	Source/rationale of recommendation	Survey rating	Participants who selected ‘do not know/no opinion’ (*n*)	Notes
			Mean (SD)		
**1**. When appropriate, future studies should ensure parallel pre‐trends (which presumes that in the absence of the exposure, the difference between the ‘exposed’ and ‘control’ groups would remain constant over time) using an event study design	Future studies should ensure parallel trend assumptions are met (namely that in the absence of the exposure, the difference between the ‘exposed’ and ‘control’ groups would remain constant over time)	Critical appraisal of studies included in review	4.1 (1.32)	8	As per survey comments, this is only applicable to some study designs, e.g. difference‐in‐differences methods Rephrased following feedback from survey Event study designs show the effect of the policy in mutually exclusive periods of time before and after the event (e.g. policy change) occurs [6, 10]
**2**. Future studies should compare outcomes of interest across different jurisdictions/contexts that vary based on a relevant exposure (e.g. comparing a province where e‐cigarettes are banned with a province where e‐cigarettes are not banned)	Same as final	Critical appraisal of studies included in review	4.43 (1.01)	0	
**3.** When relevant, future studies should investigate the possibility of dose–response effects	Future studies should investigate the possibility of dose–response effects (e.g. differences between small e‐cigarette taxes and large e‐cigarette taxes on relevant outcomes)	Critical appraisal of studies included in review	3.39 (0.90)	2	Rephrased following feedback from survey
**4.** When possible, future studies should control for other relevant policies that correlated with the policy under evaluation	Future studies should control for other relevant policies that occur simultaneously with the policy under evaluation	Critical appraisal of studies included in review	4.4 (1.22)	3	Concerns were raised that it is not always possible to evaluate individual pieces of a policy package, and interpretation should be reflected accordingly in this case Rephrased following feedback from survey
**5**. When relevant, future studies should include fixed effects for place and time over which the exposure varies to eliminate confounding from unobserved time‐invariant/area‐specific sources and area‐invariant/time‐specific sources	Future studies should include fixed effects for place and time over which the exposure varies to eliminate confounding from unobserved time‐invariant /area‐specific sources and area‐invariant/time‐specific sources	Critical appraisal of studies included in review	4.18 (1.36)	0	As flagged by one survey respondent, this depends on the study design context (e.g. may not be appropriate for a case study of a single treated jurisdiction using synthetic control methods) Rephrased following feedback from survey
**6.** Future studies should examine associations between e‐cigarette use/availability and smoking cessation in young people	Same as final	Gap in EGM	4.19 (1.24)	0	
**7**. Future studies should discuss and/or account for implementation (e.g. the time between policy enactment and effect) – including conducting sensitivity analyses to account for implementation lags – in studies where the exposure is a policy	Same as final	Critical appraisal of studies included in review	4.142 (1.29)	0	Addressed in discussion

Abbreviations: EGM, evidence and gap map; SD, standard deviation.

We then developed an anonymized online questionnaire reflecting these 22 items using Microsoft Forms (Microsoft, Redmond, WA, USA), primarily targeting researchers and policy professionals in this field. Our call for respondents was disseminated via two posts on X (formerly Twitter), through our professional networks, through the CRUK Electronic Cigarette Research Forum newsletter, and through presentations at the Tobacco Online Policy Seminar (TOPS, https://www.tobaccopolicy.org/) and at the Society for Research on Nicotine and Tobacco (SRNT) 2024 conference. Respondents were asked to rate each draft recommendation on a Likert scale from 1 (not at all important) to 5 (very important), with the option to add comments in a free‐text box for each recommendation. The survey was open between 16 February 2024 and 24 March 2024.

After the survey closed, we conducted a descriptive analysis for each item using Microsoft Excel, summarizing responses by country and stakeholder professional role. Recommendations scoring an average of ≥3 out of the maximum score of 5 were included in this definitive version of the recommendations.

### Results from the consultation exercise

Thirty‐six stakeholders responded to our survey. Twenty‐seven responded to the question about their country, with the greatest representation being from the USA (*n* = 18). Four respondents were from the UK, three were from Canada and one each were from Switzerland and Norway. All stakeholders stated their professional activity. Most respondents were researchers (*n* = 26), one was a researcher and policymaker, one was a researcher and worked in the non‐profit/charity sector, two were researchers and clinicians, two worked in the non‐profit/charity sector and the remainder consisted of one government employee, one clinician and two respondents who indicated their affiliation as ‘industry’ (see Appendix S1).

Thirty‐six respondents rated the importance of each of the 22 recommendations in the survey, with all scoring ≥3 on average. The free‐text responses also offered important additional insights. Survey results are reported in full in Appendix S1. We analysed and discussed all responses within the author team and integrated the feedback, when applicable, to the recommendations, including the addition of one new recommendation.

The resulting 23 recommendations are presented in Tables 1, 2 and 3. They are divided into three main types:

1. Recommendations relating to studies using repeat cross‐sectional data tracking population trends, encompassing studies with repeated cross‐sectional measures that evaluated combustible tobacco use in young people in relation to e‐cigarette use or availability (or both) in a given population (Table 1);
2. Recommendations relating to longitudinal cohort studies tracking behaviours in individuals, encompassing cohort studies that prospectively collected data on e‐cigarettes and combustible tobacco use from the same individuals at a minimum of two time points (Table 2);
3. Recommendations relating to both of the study types described above (Table 3).

**TABLE 2:** Longitudinal cohort studies tracking behaviours in individuals.

Final recommendation	Original recommendation	Source/rationale of recommendation	Survey rating	Participants who selected ‘do not know/no opinion’ (*n*)	Notes
			Mean (SD)		
**8.** Future studies should consider using instrumental variable designs, if an appropriate instrument is available, to identify the causal effect of vaping on subsequent smoking	Future studies should use instrumental variable designs, if an appropriate instrument becomes available, to identify the causal effect of vaping on subsequent smoking. In this study design, an instrument is a variable that conceptually impacts the outcome only through the exposure and that strongly predicts the exposure. In this context, an appropriate instrument would be a characteristic that would affect one group’s likelihood of vaping (e.g. an allergy to vegetable glycerine) but would not otherwise impact their likelihood of smoking	Critical appraisal of studies included in review	4 (1.11)	7	In this study design, an instrument is a variable that conceptually impacts the outcome through the exposure alone and that strongly predicts the exposure. In this context, an appropriate instrument would be a characteristic that would affect one group’s likelihood of vaping (e.g. an allergy to vegetable glycerine) but would not otherwise impact their likelihood of smoking A survey respondent recommended that the exclusion restriction, or the assumption that the instrument impacts the outcome only through the exposure, should be rigorously defended
**9** **.** Future studies that include people who smoke at baseline should control for combustible tobacco use at baseline, or use individual fixed effects	Future studies that include people who smoke at baseline should control for combustible tobacco use at baseline	Critical appraisal of studies included in review; recommendations for further research in review	4.51 (1.12)	2	A survey respondent noted that individual fixed effects accomplish the same goal of removing all time‐invariant individual‐level behaviours, such as baseline smoking behaviours
**10** **.** Future studies should include at least one (and ideally more than one) variable related to propensity to smoke as a covariate (for example, parental smoking, measure of susceptibility to smoking or socio‐economic status) or use individual fixed effects	Future studies should include at least one (and ideally more than one) variable related to propensity to smoke as a covariate (for example, parental smoking, measure of susceptibility to smoking or socio‐economic status)	Inclusion criteria of review	4.13 (1.19)	0	A survey respondent noted that individual fixed effects accomplish the removal of all time‐invariant individual‐level behaviours, such as baseline propensity to smoke
**11.** Future studies should report differences in missing data by exposure group	Same as final	Critical appraisal of studies included in review	4.39 (1.02)	1	
**12.** Future studies should conduct and report sensitivity analyses to test the impact of missing data	Same as final	Critical appraisal of studies included in review	4.28 (1.23)	2	This only applies where data is missing and could plausibly impact outcomes
**13.** Future studies should report the proportion of participants lost to follow‐up by exposure group	Same as final	Critical appraisal of studies included in review	4.38 (1.35)	0	
**14.** Future studies should report the proportion of participants lost to follow‐up stratified by characteristics connected to combustible tobacco use (other than the exposure of interest)	Same as final	Critical appraisal of studies included in review	4.19 (1.01)	3	

Abbreviations: EGM, evidence and gap map; SD, standard deviation.

**TABLE 3:** Recommendations for all studies.

Final recommendation	Original recommendation	Source/rationale of recommendation	Survey rating	Participants who selected “do not know/no opinion” (N)	Notes
			Mean (SD)		
**15.** Future studies should pre‐register research and/or analysis plans and/or study protocols on publicly available registers (e.g. Open Science Framework) ahead of conducting the studies, and report this in the publication	Same as final	Critical appraisal of studies included in review	4.09 (2.83)	1	Most comments were about the practicalities of doing this, especially for organizations outside of academia. We recommend analysis plans be registered in advance on free sites, such as the Open Science Framework, which is accessible to all
**16.** Future studies should use triangulation methods across a range of study designs capable of producing causal effects, but that vary in terms of internal and externality validity, to support stronger causal inference	Same as final	Recommendations for further research in review	4.24 (0.86)	0	We note (as flagged by some respondents) that this may not be feasible for some studies. It may be particularly useful in the case of evidence synthesis
**17.** Future studies should examine and report possible causes of differences in vaping–smoking transitions and associations, including sociodemographic characteristics (age, gender/sex, sexual orientation and identity, race/ethnicity, religion, occupation, socio‐economic status) and contextual factors (e.g. jurisdiction‐level policies and enforcement, public perceptions of vaping and smoking)	Same as final	Recommendations for further research in review; gap in Evidence and Gap Map	4.45 (1.11)	0	As flagged by one respondent, heterogeneity analyses should acknowledge whether the study is powered to detect effects for subgroups of interest
**18.** Future studies should generate and use representative longitudinal data from countries other than the USA, Canada and the UK, to explore the relationship between vaping and smoking in contexts that have different smoking and vaping rates among young people, and sociocultural and/or regulatory environments, particularly in low‐ and middle‐income countries and the global south	Same as final	Recommendations for further research in review; gap in Evidence and Gap Map	4.30 (1.22)	1	
**19.** Future studies should ensure that selection into their sample is not itself impacted by the exposure variable (e.g. sample is not itself endogenously impacted by the exposure variable under study), and ideally that participants are randomly selected from a national/state/province‐level representative survey or from a relevant subsample of a representative survey	Future studies should ensure that participants are randomly selected from a national/state/province‐level representative survey or from a relevant subsample of a representative survey that is itself not impacted by the exposure variable (e.g. subsample is not itself endogenously impacted by the exposure variable under study)	Critical appraisal of studies included in review	4.36 (1.21)	0	Rephrased following feedback from survey
**20.** Future studies should put in place and report on measures to ensure the anonymity of respondents, and report on the measures they undertook	Same as final	Critical appraisal of studies included in review	4.15 (1.13)	0	Authors of articles using deidentified secondary data sets do not control the conditions under which the original data set was collected, but regardless authors should state what these conditions were. Data providers should provide information on measures to ensure the anonymity of respondents in data documentation.
**21.** Future studies should follow relevant reporting guidelines, according to the type of study (e.g. The Strengthening the Reporting of Observational Studies in Epidemiology (STROBE) statement for longitudinal studies), whenever applicable	Future studies should follow relevant reporting guidelines, according to the type of study (e.g. The Strengthening the Reporting of Observational Studies in Epidemiology (STROBE) statement for longitudinal studies)	Data extraction of review	4.14 (1.24)	3	As flagged in survey responses, journal word count may prohibit this in the main text of articles, but if that is the case, supplemental material should be used to ensure reporting guidelines are adhered to
**22.** Future studies should clearly specify the frequency of vaping and smoking (e.g. experimental and regular) whether used as exposure variables or outcome variables	Same as final	Critical appraisal of studies included in review; Recommendations for further research in review	4.31 (1.54)	0	As flagged in survey responses, this may be difficult when using already existing data sets. However, authors should acknowledge this as a limitation, where relevant
**23.** Future studies should report characteristics of e‐cigarettes/electronic nicotine devices	NA – new recommendation	Survey response; gap in EGM	NA	NA	Recommendation added after consultation exercise

Abbreviations: EGM, evidence and gap map; NA, not applicable; SD, standard deviation.

We used these categories in accordance with the inclusion criteria of our review [8], which we also used in our EGM [7].

We present the rationale for each recommendation, including how we integrated respondents’ written responses in Tables 1, 2, 3. We also present the original recommendations when amendments were suggested by respondents. Nine recommendations were rephrased, and one new recommendation was added following the consultation exercise. Box 1 shows the final list of recommendations.

BOX 1 **Recommendations for future research exploring e‐cigarette use and later cigarette smoking in young people**

**1.** When appropriate, future studies should ensure parallel pre‐trends (which presumes that in the absence of the exposure, the difference between ‘exposed’ and ‘control’ groups would remain constant over time) using an event study design

**2.** Future studies should compare outcomes of interest across different jurisdictions/contexts that vary based on a relevant exposure (e.g. comparing a province where e‐cigarettes are banned with a province where e‐cigarettes are not banned)

**3.** When relevant, future studies should investigate the possibility of dose–response effects

**4.** When possible, future studies should control for other relevant policies that correlate with the policy under evaluation

**5.** When relevant, future studies should include fixed effects for place and time over which the exposure varies to eliminate confounding from unobserved time‐invariant/area‐specific sources and area‐invariant/time‐specific sources

**6.** Future studies should examine associations between e‐cigarette use/availability and smoking cessation in young people

**7.** Future studies should discuss and/or account for implementation (e.g. the time period between policy enactment and effect) – including conducting sensitivity analyses to account for implementation lags – in studies where the exposure is a policy

**8.** Future studies should consider using instrumental variable designs, if an appropriate instrument is available, to identify the causal effect of vaping on subsequent smoking

**9.** Future studies that include people who smoke at baseline should control for combustible tobacco use at baseline, or use individual fixed effects

**10.** Future studies should include at least one (and ideally more than one) variable related to propensity to smoke as a covariate (for example, parental smoking, measure of susceptibility to smoking or socio‐economic status), or use individual fixed effects

**11.** Future studies should report differences in missing data by exposure group

**12.** Future studies should conduct and report sensitivity analyses to test the impact of missing data

**13.** Future studies should report the proportion of participants lost to follow‐up by exposure group

**14.** Future studies should report the proportion of participants lost to follow‐up stratified by characteristics connected to combustible tobacco use (other than the exposure of interest)

**15.** Future studies should pre‐register research and/or analysis plans and/or study protocols on publicly available registers (e.g. Open Science Framework) ahead of conducting the studies, and report this in the publication

**16.** Future studies should use triangulation methods across a range of study designs capable of producing causal effects, but that vary in terms of internal and externality validity, to support stronger causal inference

**17.** Future studies should examine and report possible causes of differences in vaping–smoking transitions and associations, including sociodemographic characteristics (age, gender/sex, sexual orientation and identity, race/ethnicity, religion, occupation, socio‐economic status) and contextual factors (e.g. jurisdiction‐level policies and enforcement, public perceptions of vaping and smoking)

**18.** Future studies should generate and use representative longitudinal data from countries other than the USA, Canada and the UK, to explore the relationship between vaping and smoking in contexts that have different smoking and vaping rates among young people, and sociocultural and/or regulatory environments, particularly in low‐ and middle‐income countries and the global south

**19.** Future studies should ensure that selection into their sample is not itself impacted by the exposure variable (e.g. sample is not itself endogenously impacted by the exposure variable under study), and ideally that participants are randomly selected from a national/state/province level representative survey or from a relevant subsample of a representative survey

**20.** Future studies should put in place and report on measures to ensure the anonymity of respondents, and report on the measures they undertook

**21.** Future studies should follow relevant reporting guidelines, according to the type of study (e.g. The Strengthening the Reporting of Observational Studies in Epidemiology (STROBE) statement for longitudinal studies), whenever applicable

**22.** Future studies should clearly specify the frequency of vaping and smoking (e.g. experimental and regular), whether used as exposure variables or outcome variables

**23.** Future studies should report characteristics of e‐cigarettes/electronic nicotine devices

## DISCUSSION

To the best of our knowledge, this work is the first attempt to produce a set of recommendations to guide areas of improvement in quantitative research looking at e‐cigarette use and later smoking in young people. Drawing from key findings of an EGM, a systematic review, a consensus setting exercise and in‐depth discussion within the research team, we produced a set of 23 recommendations. The recommendations are divided into three segments representing the study types we found in our previous research [7, 8]. They do not intend to mandate strict requirements for future studies but rather offer broad guidance that should be interpreted through the specific lens of the work being planned. We acknowledge that these recommendations may not cover all aspects relevant to possible valid study designs, particularly best approaches for drawing causal inference [3], as this is a complex area of continuous methodological development, which was outside the scope of this work. Other recent articles provide further suggestions on how best to establish causality from observational data [11, 12]. Further priorities may be identified through other research, for example, recommending sample sizes and/or power analyses for longitudinal studies [13]. Nonetheless, it was reassuring that the recommendations were considered important by expert stakeholders in this research area. Stakeholders’ constructive comments also offered important insights that refined this work.

We also acknowledge difficulty in organizing recommendations by data type, as quasi‐experimental analysis can be performed within any type of data. For example, although we have a recommendation concerning instrumental variable (IV) techniques for studies using individual‐level longitudinal data, IV models can also be performed using cross‐sectional data. Similarly, difference‐in‐differences models can be estimated with individual‐level data. This highlights a potential need to reconsider data type as an organizing principle for research. Moreover, research teams should ensure robust statistical support when conducting these studies.

Most respondents were researchers from the USA, followed by the UK and Canada. This pattern aligns with the current geographical distribution of studies, with evidence predominantly originating from the USA [7]. Evidence is lacking from global regions with the greatest burden of tobacco‐related diseases and mortality [1, 14, 15]. Most available research consists of secondary analyses from existing longitudinal data sets, which may limit the types of data that researchers can access – particularly sociodemographic factors relevant to equity considerations [16]. A lack of evidence on potential differences by gender and sex in transitions between smoking and vaping illustrates this point [17]. These aspects should be considered when planning and setting up new longitudinal studies. There are still areas for improvement in high‐income countries, including the representativeness of the samples included. We developed our recommendations keeping the limitations of the existing literature in mind.

We recognize that some of our recommendations may already be covered by existing reporting guidelines in health research, such as the Strengthening the Reporting of Observational Studies in Epidemiology (STROBE) statement [18]. However, as STROBE guidelines may not be appropriate (or followed) for all studies in this area, we pulled out the elements that emerged as critical in our review and EGM. Moreover, STROBE is discipline specific, and other tools [19] may be more appropriate for quasi‐experimental research. Nonetheless, most of our recommendations for this area of research are specific to studies using repeat cross‐sectional data tracking population trends and to longitudinal cohort studies tracking behaviours in individuals. Even though respondents globally supported pre‐registering analysis and study plans, highlighting the importance of transparency, there remains room for greater consensus within the research community. Context‐specific barriers and concerns about the practicality of pre‐registration for secondary analyses were conveyed in the variation of responses for recommendation 15.

Furthermore, we appreciate the complexity of the interactions between research and real‐world implementation. The underlying drivers of policy landscapes are context specific and will vary by country. Therefore, some recommendations may need to be adapted depending on national policy, regulatory environments (e.g. use of policy effective dates) and sociocultural setting (e.g. religious framework, people's and businesses’ behaviours prior to the implementation of an announced policy, different confounding factors, etc.).

This work has limitations. The relatively low number of respondents, with most being from the USA, may have narrowed the range of perspectives captured. However, this coincides with the profile of current research in the field. The possibility of selection bias and other stakeholder engagement biases [20] needs to be considered. Furthermore, the survey items were not randomized, which may contribute to fatigue effects and order effects. However, all participants completed all Likert scale items of the survey. Our recommendations were originally derived from methodologically robust evidence mapping and synthesis following the best standards of research [7, 8]. Our team consists of methodologists and researchers in e‐cigarettes and smoking – covering public health, policy and economic expertise – to interpret the quality and gaps in the evidence base. Nevertheless, there are still limitations inherent to those processes that may have been reflected in this work.

This area of knowledge embeds health, policy and economic research. There are opportunities to foment further cross‐disciplinary collaboration to enhance reporting standards, thereby improving the quality and transparency of the research exploring this problem. These recommendations can hopefully promote constructive interdisciplinary discussions.

## CONCLUSION

Our set of recommendations can support researchers in conducting and reporting future quantitative research exploring e‐cigarette use/availability and later combustible cigarette smoking in young people. These recommendations were derived from rigorous evidence mapping and synthesis, together with input from expert stakeholders. They represent a first step in addressing some of the key methodological issues found in current research, which may contribute to improving the quality and transparency of the primary evidence needed to support evidence synthesis and policy‐ and decision‐making in this field. Future research could also explore the real‐world impact of these recommendations, and how researchers should best communicate complex findings to decision makers.

## AUTHOR CONTRIBUTIONS

**Monserrat Conde:** Conceptualization (equal); data curation (lead); formal analysis (equal); investigation (equal); methodology (equal); project administration (lead); validation (lead); visualization (lead); writing—original draft (lead); writing—review and editing (lead). **Michael F. Pesko:** Formal analysis (equal); investigation (supporting); methodology (supporting); validation (equal); writing—review and editing (supporting). **Lion Shahab:** Formal analysis (equal); investigation (supporting); methodology (supporting); validation (equal); writing—review and editing (supporting). **Rachna Begh:** Formal analysis (equal); investigation (supporting); methodology (supporting); validation (equal); writing—review and editing (supporting). **Nicola Lindson:** Formal analysis (supporting); funding acquisition (supporting); investigation (supporting); methodology (supporting); validation (supporting); writing—review and editing (supporting). **Sarah E. Jackson:** Formal analysis (supporting); investigation (supporting); methodology (supporting); validation (supporting); writing—review and editing (supporting). **Dimitra Kale:** Formal analysis (supporting); investigation (supporting); methodology (supporting); validation (supporting); writing—review and editing (supporting). **Dylan Kneale:** Formal analysis (supporting); investigation (supporting); methodology (supporting); validation (supporting); writing—review and editing (supporting). **Jonathan Livingstone‐Banks:** Formal analysis (supporting); investigation (supporting); methodology (supporting); validation (supporting); writing—review and editing (supporting). **Jamie Hartmann‐Boyce:** Conceptualization (lead); data curation (supporting); formal analysis (equal); funding acquisition (lead); investigation (supporting); methodology (equal); project administration (supporting); supervision (lead); validation (equal); writing—original draft (supporting); writing—review and editing (supporting).

## DECLARATION OF INTERESTS

The work of M.C. is funded by Cancer Research UK (PPRCTAGPJT\10 000). This was not deemed a conflict of interest. The work of M.P. is funded by the National Institute on Drug Abuse of the National Institutes of Health (R01DA045016). M.P. declares current funding from the Food and Drug Administration, the American Cancer Society, University of Kentucky Institute for the Study of Free Enterprise and Health Canada. L.S. is a Higher Education Funding Council for England (HEFCE) funded member of staff at University College London. He has received honoraria for talks, an unrestricted research grant and travel expenses to attend meetings and workshops from Pfizer, and an honorarium to sit on an advisory panel from Johnson & Johnson, both of which are pharmaceutical companies that make smoking cessation products. He has acted as paid reviewer for grant awarding bodies and as a paid consultant for healthcare companies. Other research has been funded by the Department of Health, UK Research and Innovation, a community‐interested company (National Centre for Smoking Cessation) and charitable sources (Cancer Research UK, Yorkshire Cancer Research). He has never received personal fees or research funding of any kind from alcohol, e‐cigarette or tobacco companies. R.B. has no competing interests to report. N.L. is Associate Editor at *Addiction*, with no other competing interests. S.J. is a Senior Editor at *Addiction*, with no other competing interests. Dimitra Kale is an associate editor at *Addiction*, with no other competing interests.

Dylan Kneale has no competing interests to report. J.L.B. has no competing interests to report. J.H.B. declares support from the Truth Initiative and the US Food and Drug Administration on work related to tobacco control and e‐cigarettes. She receives research grants from the National Institutes of Health, the US Food and Drug Administration and Cancer Research UK.

## Supporting information


**Appendix S1.** Supplementary information.

## Data Availability

Data available in article supplementary material.
