# Supplementary material for: Recommendations for future research exploring e‐cigarette use and later cigarette smoking in young people: Results from a consultation exercise
Source: Addiction. 2025 Mar 5;120(8):1679–88. doi: 10.1111/add.70038 (PMC12215211; doi:10.1111/add.70038)
Supplement: Supplementary file 1 — Appendix S1. Supplementary information. [file ADD-120-1679-s001.docx]

Contents

[Rationale the call for respondents 1](#_Toc181992096)

[Results from the consultation exercise (online survey) 2](#_Toc181992097)

[Characteristics of stakeholders 2](#_Toc181992098)

[Feedback on original recommendations 2](#_Toc181992099)

[Studies using repeat time cross-sectional data tracking population trends 12](#_Toc181992100)

[Longitudinal cohort studies tracking behaviours in individuals 19](#_Toc181992101)

[Open question 25](#_Toc181992102)

[Open question: “Please provide any other overall comments or suggestions you may have.” 25](#_Toc181992103)

[Process for integrating the feedback from responders into the final recommendations 26](#_Toc181992104)

# Rationale the call for respondents

Obtaining relevant input from key stakeholders is critical to developing useful recommendations for research. We primarily targeted researchers and policy professionals working in this field. Researchers have in-depth methodological knowledge and experience in conducting studies, as well as a comprehensive understanding of the state of the evidence and existing gaps. Policy professionals have a key role in transforming research evidence into action, as well as a profound understanding of the priority areas for decision-making in specific policy contexts. Combining the input of these groups could ensure that our recommendations were realistic, fit-for-purpose and could lead to constructive change. However, we also recognized the relevance of other knowledge users (e.g., clinicians). Therefore, we did not set rigid limits regarding the types of stakeholders.

We adopted a pragmatic approach and disseminated our call for respondents at major scientific meetings that could be attended by our target stakeholders. We did not exclude respondents based on professional context since research on this topic area is commissioned and conducted in a wide range of settings.

## Results from the consultation exercise (online survey)

Please note that this document follows the same order as the questions of the online survey. The order of the original recommendations was as follows: all studies, studies using repeat time cross-sectional data tracking population trends**, l**ongitudinal cohort studies tracking behaviours in individuals.

### Characteristics of stakeholders

Thirty-six stakeholders responded to our survey. Twenty-seven responded to the question about their country, with the greatest representation being from the USA (n=18). Four respondents were from the UK, three from Canada and one from Switzerland and from Norway, respectively. All 36 stakeholders stated their professional activity. Most respondents were researchers (n=26), one was a researcher and policymaker, one was a researcher and worked in non-profit/charity sector; two were researchers and clinicians; two worked in the nonprofit/charity sector, and the remainder consisted of one government employee, one clinician, one respondent who indicated their affiliation as ‘industry’ and another as ’consultant in industry’.

### Feedback on original recommendations

**Part 1 - All studies**

**Recommendation 1.** *Future studies should pre-register research and/or analysis plans and/or study protocols on publicly available registers (e.g. Open Science Framework) ahead of conducting the studies, and report this in the publication.*

Fifteen stakeholders considered that this recommendation was “very important”, followed by 13 that considered it “somewhat important.”


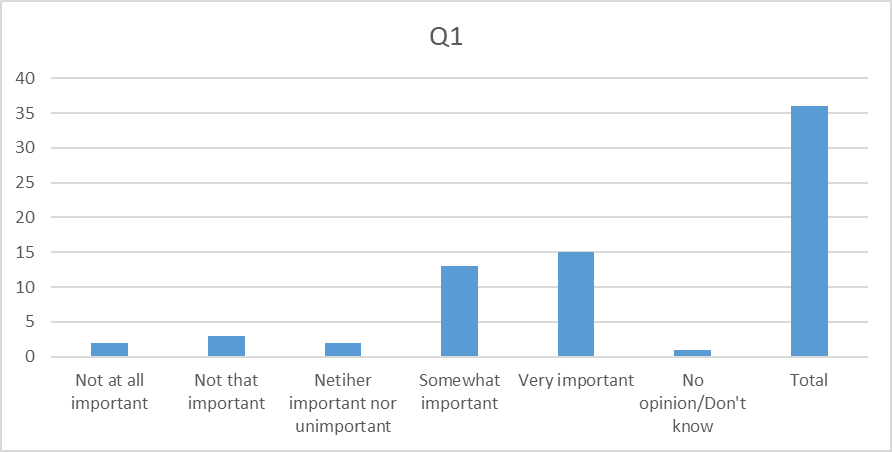


**Open question for recommendation 1**

We had seven responses to this open question.

“While pre-registration is not a guarantee of reliable results, it does increase the ability of the reader to evaluate reliability.”

“This question is too vague. For randomized trials, pre-registration is very important. For observational studies, pre-registration is not important. Also, pre-registration is different than pre-analysis plans. My view is that pre-analysis plans can be too binding.”

“I agree that this is optimal and should be 'gold standard' but pragmatically some organisations working outside the academic space may struggle to do this plus sometimes datasets may become available opportunistically etc. That doesn't necessarily invalidate their evidence but certainly greater weight should be afforded to those with a stronger process and a pre-registering seems an important part of this.”

“Pre-registering is standard for RCTs but at least as important when it comes to secondary data analyses for which choices in design and analysis can be determinate.”

“Note that this is very important where applicable. For secondary data studies, it is sometimes not possible to follow a pre-registered plan until you see the data because of e.g., sample size issues. Also in large data sets that are regularly used, researchers often know a lot about the data already, so it's had to say that they haven't looked at the data prior to analysis.”

“Absolutely essential to avoid 'fishing exercises'. Many journals are now requiring this and others ask for a statement about pre-registration to be included.”

“Registration is important both for transparency and for enabling research to go forward which is complementary to existing research.”

**Recommendation 2**

*Future studies should use triangulation methods (Consider data from multiple methodological approaches, each with different sources of bias*) across a range of study designs capable of producing causal effects, but that vary in terms of internal and externality validity, to support stronger causal inference.*

**in Lawlor DA, Tilling K, Davey Smith G. Triangulation in aetiological epidemiology. Int J Epidemiol. 2016;45(6):1866–1886.*

Nineteen respondents considered that this recommendation was “somewhat important”, followed by 15 that considered it “very important.


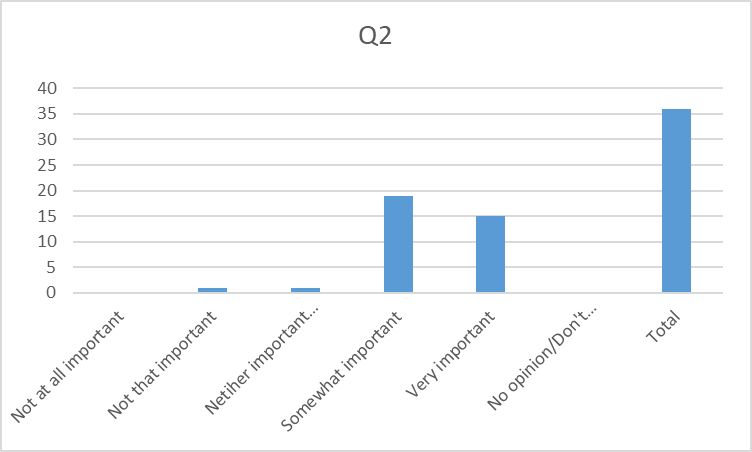


**Open question to recommendation 2**

We had 10 responses to this question.

“I don't think every study needs to triangulate, but policy or clinical conclusions need to.”

“This makes the research exercise harder, particularly as our minds are configured to remember/attend to information that conforms to our priors and to "forget" information that does not--so being tasked with integrating a wider evidence base increases this risk and thus the ideal that research teams reflect as much viewpoint diversity as possible. To that point, you might consider the utility of engaging with this initiative: <https://web.sas.upenn.edu/adcollabproject/> “

“If used across designs that can produce causal effects under testable assumptions, where those assumptions are indeed tested, I would find this extremely important. However, I worry that the definition of triangulation is too broad: I’ve reviewed multiple papers where researchers argue that a causal conclusion is warranted because findings are consistent across studies using multiple approaches, ignoring the fact that all of those approaches are subject to the same bias in the direction of the estimated effect.”

“Replicating results with additional sources of data is less important than the strength of the empirical design. “

“This would be optimal but not all studies will have access to funding to enable triangulation. It may still be useful to have data in peer review.”

“I've answered 'very important' but to be honest this is something that is very important for evidence reviews to do as opposed to individual studies.”

“No one can study can prove a theory and doing the same thing again and again could lead to consistent wrong answers.”

“Whilst ideal, it's not possible for all studies to do this due to limited resources but findings across studies can be triangulated.”

“This is important because, in recent years, the definition of causality appears to be more flexible than previously, a trend which can undercut good science.”

“It's a really frequentist approach to mandate this. Truth is most causal inference models are likely to be altered by modeling issues and some covariates might not be able to be analyzed because of missing or interactions might explain better the found estimands.”

**Recommendation 3**

*Future studies should examine and report possible causes of differences in vaping-smoking transitions and associations, including sociodemographic characteristics (age, gender/sex, sexual orientation and identity, race/ethnicity, religion, occupation, socioeconomic status) and contextual factors (e.g. jurisdiction-level policies and enforcement, public perceptions of vaping and smoking).*

Twenty-five respondents considered that this recommendation was “very important”, followed by eight that considered it “somewhat important.”


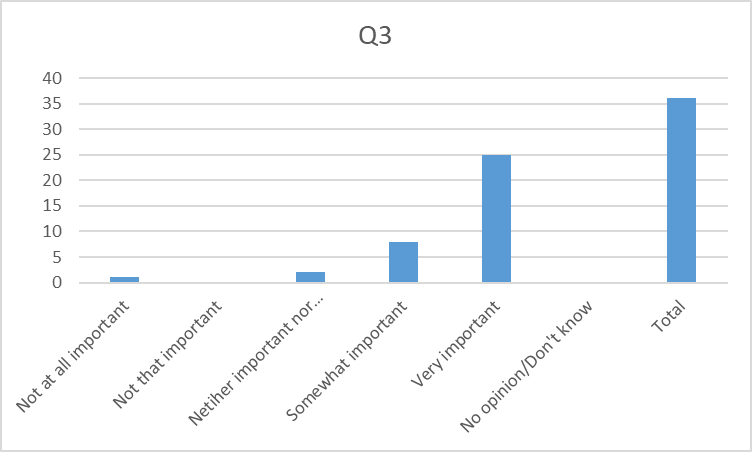


**Open question to recommendation 3**

We had five responses.

“Heterogeneity analyses should acknowledge whether the study is powered to detect an effect for certain sub-samples, whether heterogeneous effects are statistically different from each other (e.g. male vs female), and where appropriate the study should discuss how the results align with our priors (e.g. whether we have a basis to predict a larger effect for one subsample than another).”

“This is an absolute priority for further research and enriching our understanding”

“Without an understanding of the mechanistic pathways, the associations are of limited use.”

“Important for contextualization.”

**“**Very difficult to study transitions in a causal sense. We have a hard enough time modelling current use causally, let alone trying to figure out what use might be at some point in the future.”

**Recommendation 4**

*Future studies should generate and use representative data from countries other than the USA, Canada and UK, to explore the relationship between vaping and smoking in contexts that have different smoking and vaping rates among young people, and sociocultural and/or regulatory environments, particularly in low- and middle-income countries and the global south.*

Twenty-two respondents considered that this recommendation was “very important”, followed by 10 that considered it “somewhat important.”


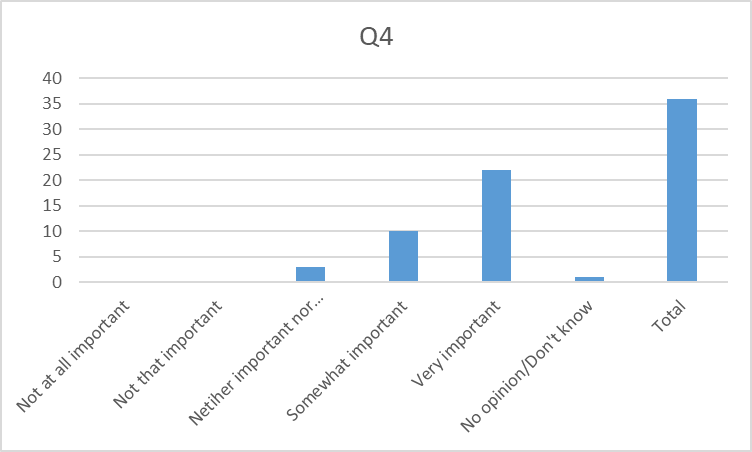


**Open question to recommendation 4**

We had three open responses

“34 countries in LMIC banned e-cig, how research can explore this is a question. Also, funding cohort research in LMIC is a challenge.”

“I think this greatly depends on the question you are looking to answer. It is right to say that generalising from the impact of vaping in one country to others, particularly those with different levels of income, policy frameworks and cultural contexts will have limited value. So if you are only interested in one country insights from elsewhere may not be useful.”

“Most tobacco/nicotine users do not live in those three countries, and research from those three countries may not apply to other country contexts.”

**Recommendation 5**

*Future studies should ensure that participants are randomly selected from a national/state/province level representative survey or from a relevant subsample of a representative survey that is itself not impacted by the exposure variable (e.g., subsample is not itself endogenously* impacted by the exposure variable under study).*

Twenty-three respondents considered that this recommendation was “very important”, followed by 10 that considered it “somewhat important.”


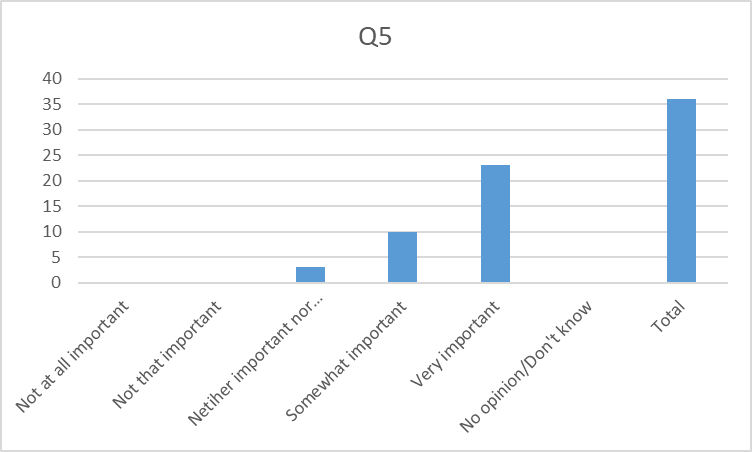


**Open question to recommendation 5**

We had four responses.

**“**This recommendation combines two recommendations. The first recommendation is that participants should be drawn from a representative sample. This is only somewhat important. A research design with strong internal validity can contribute valuable evidence on causality even if the data are not from a representative sample. The second recommendation is that selection into the sample isn't impacted by the exposure variable. This is very important for internal and external validity.”

“For qualitative research this may prove more challenging”

“Potential to control for these within statistical analyses.”

“Nice to have, reality hits hard once you need to do it. Certainly a quality criteria, I don't think they SHOULD ensure this.”

**Recommendation 6**

*Future studies should put in place and report on measures that ensure the anonymity of respondents, and report on the measures they undertook.*

Seventeen respondents considered that this recommendation was “very important”, followed by nine that considered it “somewhat important.”


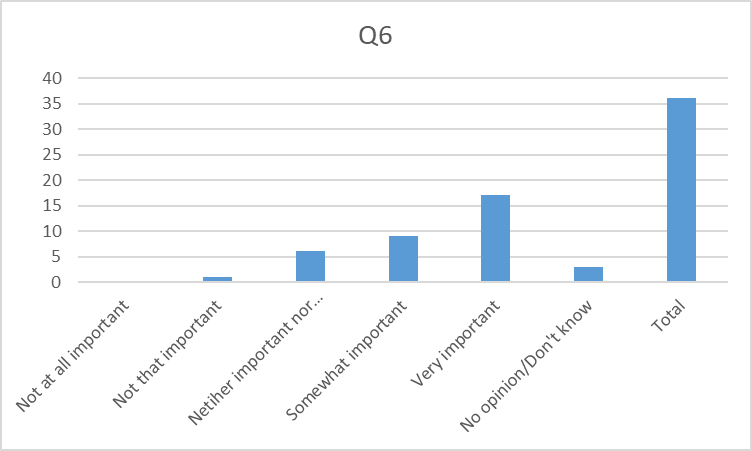


**Open question to recommendation 6**

We had six responses.

“This seems to be an already-established standard but perhaps I am wrong?”

“This is not necessary with deidentified secondary datasets that are described as such, but is certainly important in other cases.”

“Study should follow IRB guidelines.”

“This is already a requirement for academic research. I do not see this as a major priority.”

“I think that this is routinely done.”

“This is handled by Ethics committees. I don't see the point of requiring this here.”

**Recommendation 7**

*Future studies should follow relevant reporting guidelines, according to the type of study (e.g., The Strengthening the Reporting of Observational Studies in Epidemiology (STROBE) statement for longitudinal studies).*

Seventeen respondents considered that this recommendation was “very important”, followed by 12 that considered it “somewhat important.”


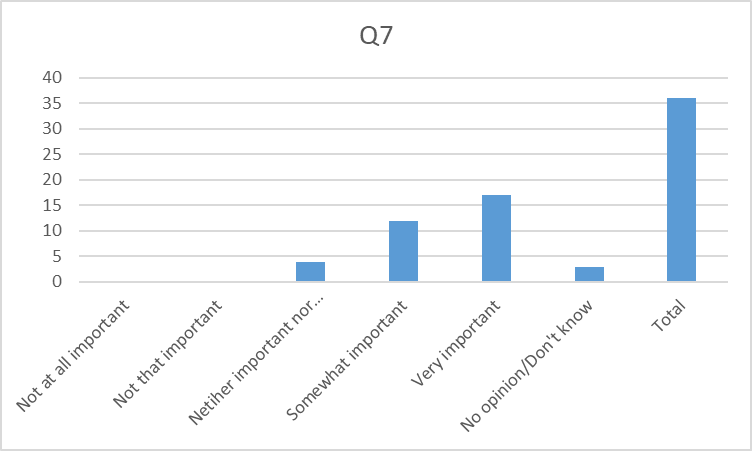


**Open question to recommendation 7**

We had five responses.

“Some of these reporting requirements are less applicable to secondary data analyses than reports of primary data collection and make it difficult to mean word limits in brief form articles. Parring the in-text reporting requirements down and adding a standardized appendix structure for studies based on primary data collection could reduce the word count burden and allow for more detail.”

“I am not familiar with these guidelines.”

“This is particularly important for research published outside of clinical and public health journals, e.g., in economics journals.”

“Important to ensure all relevant material has been included and to make comparisons across studies.”

“It's sometimes difficult to pigeon-hole quasi-experimental work into these guidelines.”

**Recommendation 8**

*Future studies should clearly specify the frequency of vaping and smoking (e.g., experimental and regular) whether used as exposure variables or outcome variables.*

Thirty-one respondents considered that this recommendation was “very important”, followed by five that considered it “somewhat important.”


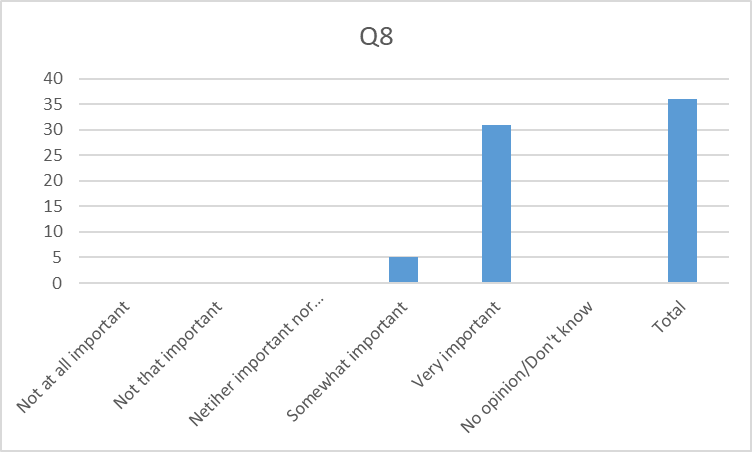


**Open question to recommendation 8**

We had 6 responses.

“Wherever possible subject to data limitations.”

“It would be great to have a more standardised metric of regular, experimental etc for both vaping and smoking to enable better comparison between studies”

“The measure clearly matters. However, this recommendation is redundant with the one above to follow STROBE guidelines. It might be belong as a corollary of the STROBE recommendation.”

“It's so important to report frequency (% and n). Many studies make bold conclusions based on a handful of people. Clearly defining the exposure is also necessary.”

“Extremely important as many studies have used 'any e-cigarette use' as predictors of later (any) smoking which is not meaningful.”

“Important, but again, I think that this is routinely done.”

## Studies using repeat time cross-sectional data tracking population trends

**Recommendation 9**

*Future studies should ensure parallel trends assumptions are met (namely that in the absence of the exposure, the difference between “exposed” and “control group” would remain constant over time).*

Fifteen respondents considered that this recommendation was “very important”, followed by nine that considered it “somewhat important.”


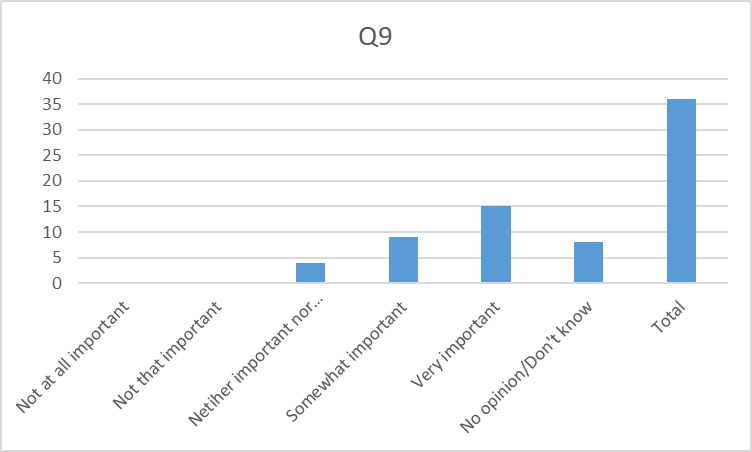


**Open question to recommendation 9**

We had 9 responses.

“This recommendation is very important for certain econometric studies that use quasi-experimental methods with observational data to draw causal inferences. It is a necessary but not sufficient condition for plausible causal inference. Of course, the recommendation is not relevant or important in a research design that uses data from a true randomized experiment. It also does not necessarily apply to some econometric quasi-experimental designs, e.g. regression discontinuity.”

“Event study design should be included and new DID methods should be used or there should be a discussion why they are not used.”

“I do not like the wording of this item. The parallel trends assumption cannot be directly tested. One can only test for parallel pre-trends.”

“I'm not sure what this would look like. Isn't this a counter-factual? How do we ensure the assumption is met? Should this be that we should state that this is an assumption of the work? I may have misunderstood, so this could be clearer if I have.”

“This entirely depends on the methodology. Synthetic difference-in-differences allows one to relax this assumption, for example.”

**Recommendation 10**

*Future studies should compare outcomes of interest across different jurisdictions/contexts that vary based on a relevant exposure (e.g. comparing a province/geographical area where e-cigarettes are banned to a province/geographical area where e-cigarettes are not banned)*.

Twenty-three respondents considered that this recommendation was “very important”, followed by 11 that considered it “somewhat important.”


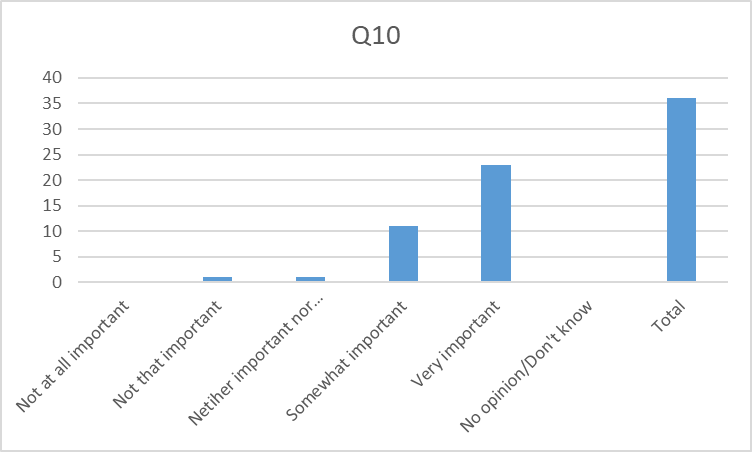


**Open question to recommendation 10**

We had seven responses.

“I think that there remains quite a bit of opportunity here. Betraying my provincial USA bias, but, for example, taking advantage in differences in CIGARETTE tax/price across US states might inform analysis of transitions: is conversion to smoking more likely in states where cigarette price is lower?”

“This can be a powerful research design with observational eventdata but it is not the only valid approach.”

“We have done quite a lot of these studies in the past. I think the literature is becoming saturated with these.”

“Depends on the questions want to answer”

“I think that the answer to this depends on whether a study includes multiple time points to assess trends.”

“Yes, there needs to be a well defined counterfactual.”

“Important for real-world insights”

**Recommendation 11**

*Future studies should investigate the possibility of dose-response effects (e.g. differences between small e-cigarette taxes and large e-cigarette taxes on relevant outcomes).*

Nineteen respondents considered that this recommendation was “very important”, followed by 10 that considered it “somewhat important.”


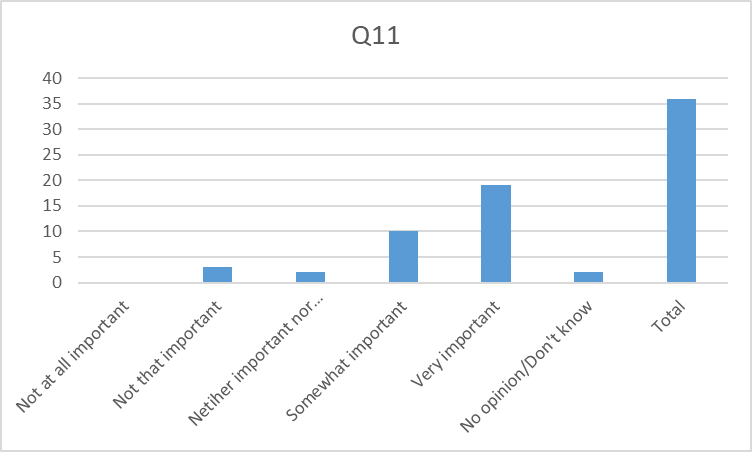


**Open question to recommendation 11**

We had three responses.

“Definitely don't think dose response is first-order concern for taxation. I think dose response is important for understanding vaping/smoking's health harms, but that is not what this question is about.”

“Dose-response relationships are important for considering causality but I'm unsure how this could be done practically in population-level studies. Exposures such as tax levels will vary by geographical area, which also introduces other differences/potential confounders.”

“Some policies do not have obvious doses.”

**Recommendation 12**

*Future studies should control for other relevant policies that occur simultaneously with the policy under evaluation.*

Twenty-three respondents considered that this recommendation was “very important”, followed by nine that considered it “somewhat important.”


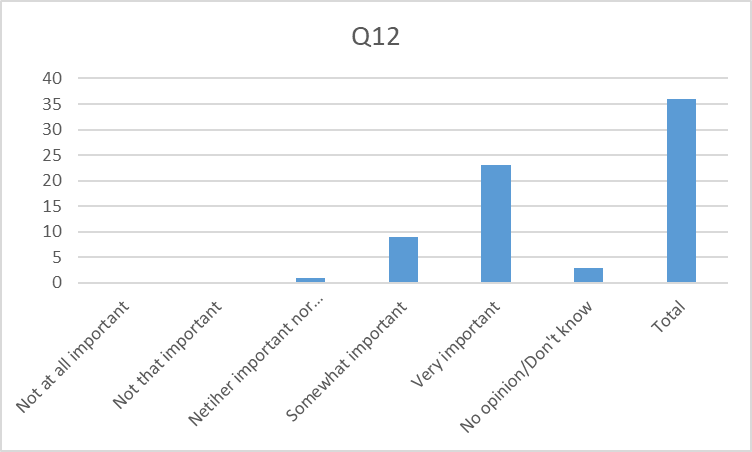


**Open question to recommendation 12**

We had six responses.

“Poor question wording. If they truly occur simultaneously, they cannot be controlled for.”

“When able to disentangle between different components of policies or components of a policy package, the study should of course do so, but it's also acceptable to acknowledge if study is unable to do so and simply provide evidence of a combined effect. Sources of confounding can also be addressed through additional fixed effects or linear unit-specific trends.”

“Though always challenging in practice”

“Again, practically - how would this be possible?”

“It is not always possible to "control" for concurrently adopted policies, depending on the context. It would be more feasible to consider them in the analysis and interpretation.”

“Not sure how this could be done.”

**Recommendation 13**

*Future studies should include fixed effects for place and time over which the exposure varies to eliminate confounding from unobserved time-invariant / area-specific sources, and area-invariant / time-specific sources.*

Sixteen respondents considered that this recommendation was “very important”, followed by seven that considered it “somewhat important.”


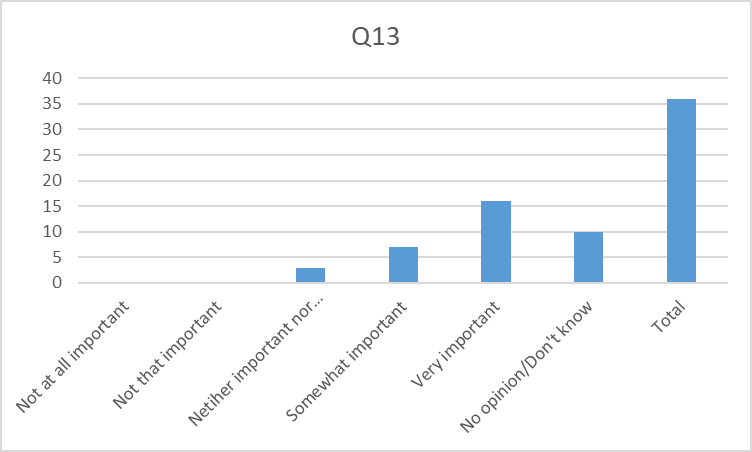


**Open question to recommendation 13**

We had one response.

“A two-way fixed effects approach is appropriate for many study designs, but may not be universally appropriate or feasible. A case study of a single treated jurisdiction that uses synthetic control methods will often not include unit or time fixed effects, but it can still be a valid study design for drawing causal inferences.”

**Recommendation 14**

*Future studies should examine associations between e-cigarette use/availability and smoking cessation in young people.*

Nineteen respondents considered that this recommendation was “very important”, followed by 14 that considered it “somewhat important.”


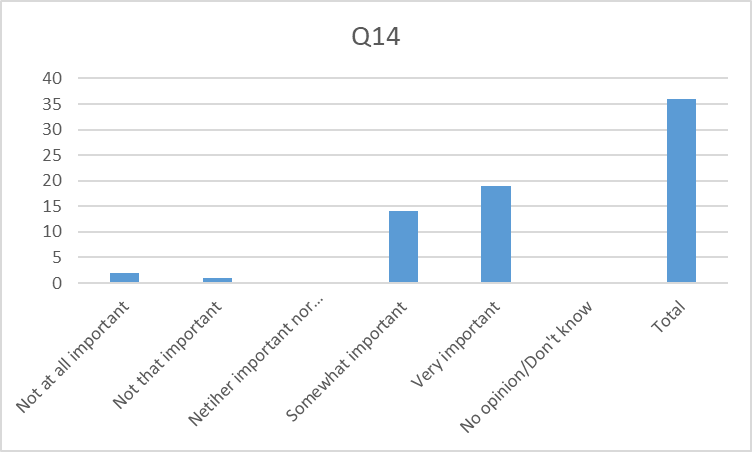


**Open question to recommendation 14**

We had three responses.

“I 100% agree with the spirit of this question, and of this research team's agenda. Cross-sectional correlations between vaping and smoking are NOT useful for telling us about the causal impact of vaping on smoking behaviors!”

“This is consistent with the recommendation of triangulation above.”

“Not sure what is meant by associations. Studying causal relationships is indeed important, but associational relationships are often highly misleading. "Smoking prevention" is just as important of an outcome as "smoking cessation" for young people.”

**Recommendation 15**

*Future studies should discuss and/or account for implementation (e.g., the time period between policy enactment and effect) - including conducting sensitivity analyses to account for implementation lags - in studies where the exposure is a policy.*

Sixteen respondents considered that this recommendation was “very important” and sixteen considered it “somewhat important.”


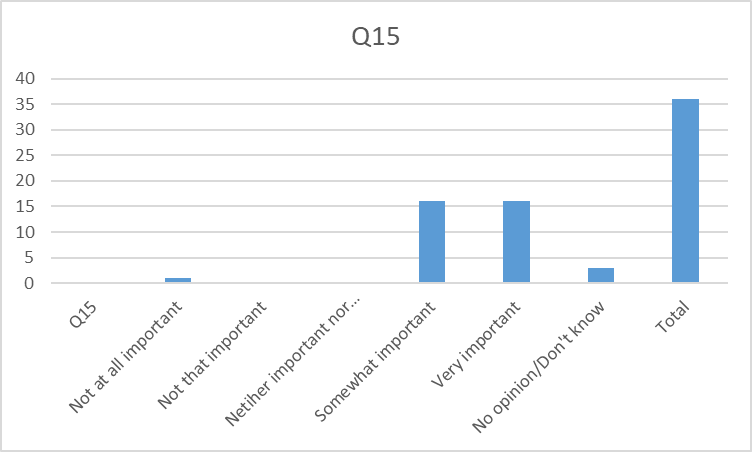


**Open question to recommendation 15**

We had four responses.

“An event study analysis is the appropriate place to discuss a lagged effect of policy, e.g. if the policy takes time to be implemented.”

“There can be a lot of complexity and understand the real world implementation landscape matters. Planned legislative changes can effect behaviour of businesses and consumers before implementation, can fail to be implemented at all etc.”

“Also policy announcement if possible. People will often change their behaviour prior to the implementation e.g., by stockpiling products or quitting / finding alternatives ahead of time.”

“Implementation time period is often endogenous to the policy. It often reflects legislators and police force's willingness to enforce the law. Further, "implementation" is usually not black and white. In some cases, people try to enforce the laws in the beginning, only to realize the public isn't buying on and then they back off without changing the law per say. So implementation is difficult to define and understand the drivers of. Policy effective dates, in contrast, are only driven by one thing: legislation. So that's more easily understandable, and easier to study free of confounding.”

## Longitudinal cohort studies tracking behaviours in individuals

**Recommendation 16**

*Future studies should use instrumental variable designs, if an appropriate instrument becomes available, to identify the causal effect of vaping on subsequent smoking. In this study design, an instrument is a variable that conceptually impacts the outcome only through the exposure and that strongly predicts the exposure. In this context, an appropriate instrument would be a characteristic that would affect one group’s likelihood of vaping (e.g. an allergy to vegetable glycerine) but would not otherwise impact their likelihood of smoking.*

Fifteen respondents considered that this recommendation was “somewhat important”, followed by 10 that considered it “very important.”


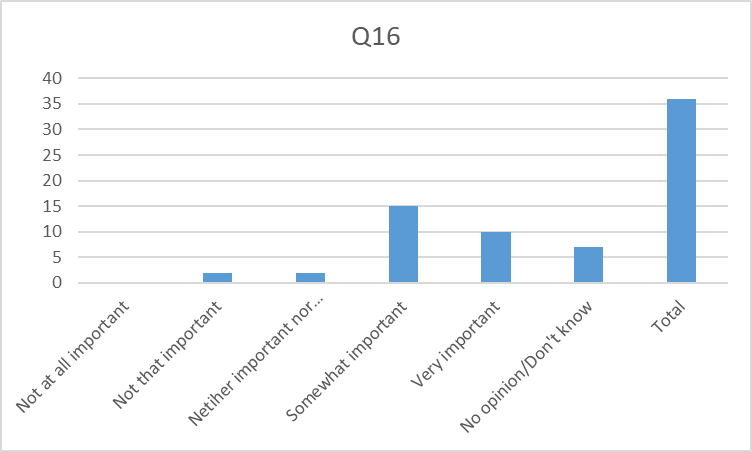


**Open question to recommendation 16**

We had six responses.

“Sounds like a beautiful instrument. Yes, someone should do that study!”

“IV results should be paired with OLS results to assess the size and direction of bias. The exclusion restriction should be defended vigorously.”

“Sounds great (and part of triangulation) but actually we don't currently know of an instrumental variables for ecigarette use.”

“IV designs are great if done well, but misleading if done poorly. I believe it is overly strong to frame the recommendation as future studies "should use" IV.”

“IV models can be useful for sure, but other quasi-experimental designs are available as well.”

“THAT'S THE MOST IMPORTANT. Modeling methods for causal inference is the key. I'm suprized it was not listed in the quality critieria. A regression models is NOT enough. It's all about modeling the probability of exposure.”

**Recommendation 17**

*Future studies that include people who smoke at baseline should control for combustible tobacco use at baseline.*

Twenty-six respondents considered that this recommendation was “very important”, followed by six that considered it “somewhat important.”


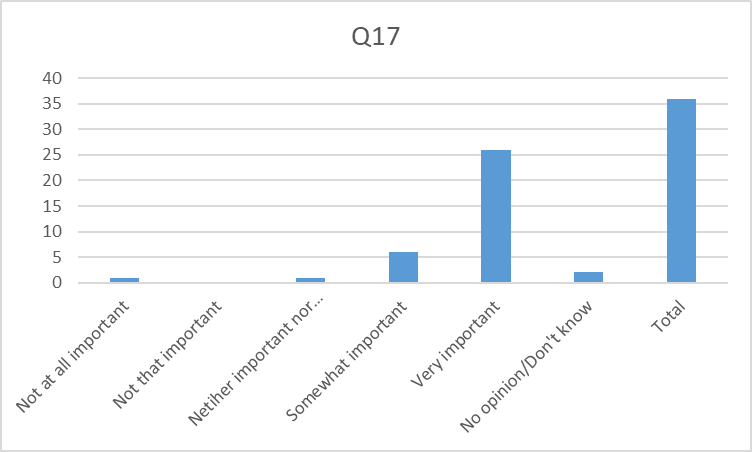


**Open question to recommendation 17**

We had three responses.

“I think there would be problems with this approach. We need to be able to better estimate the probability of exposure.”

“I don't think the studies/analyses should include people who smoke at baseline at all”

“Person fixed effect models control for the same thing in a more rigorous way, so that would be stronger.”

**Recommendation 18**

*Future studies should include at least one (and ideally more than one) variable related to propensity to smoke as a covariate (for example, parental smoking, measure of susceptibility to smoking, or socioeconomic status).*

Seventeen respondents considered that this recommendation was “very important”, followed by 15 that considered it “somewhat important.”


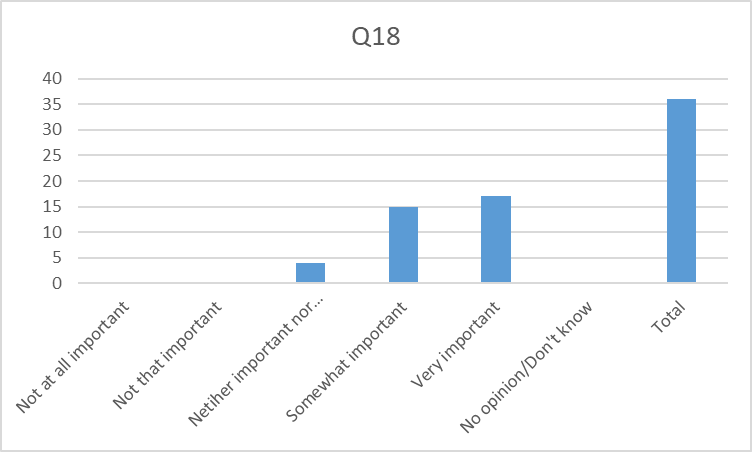


**Open question to recommendation 18**

We had three responses.

“Adjustment for socioeconomic status via complete education (or parental completed education for youths) is particularly important here, alongside age group (e.g., distinguishing 12 year-olds and 17 year-olds)”

“I think this depends on the context and full study design.”

“Again, person fixed effect models when data is available as a panel is preferred because it captures this and all other time-invariant characteristics. However, these variables can be useful, provided they are measured in the baseline period. It would be incorrect to include a time-varying version of these variables in models since the policy being studied could also affect a time-varying version of these variables.”

**Recommendation 19**

*Future studies should report differences in missing data by exposure group.*

Twenty-one respondents considered that this recommendation was “very important”, followed by 12 that considered it “somewhat important.”


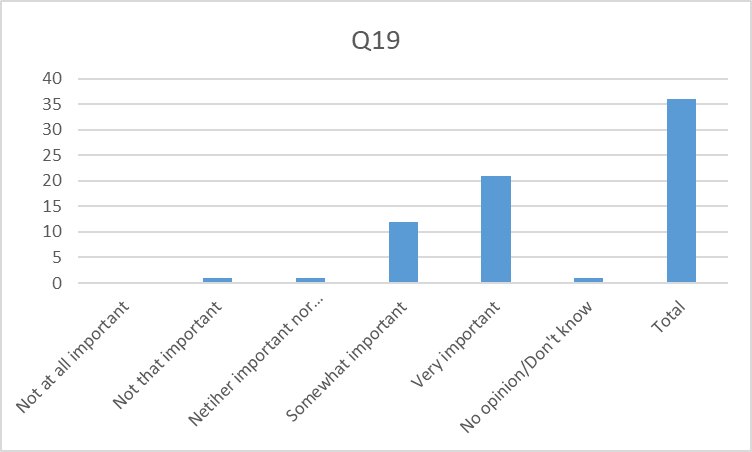


**Open question to recommendation 19**

We had four responses.

“To me, the importance of reporting these differences hinges on whether the exposure per se could impact missingness (or vice versa).”

“This is important, as is any potential source of bias from survey data, such as attrition.”

" Important, but also covered by STROBE guidelines.”

“This is important and is rarely done.”

“Most important would then be to use causal inference methods for missings. MSM could be a method. IPCW enable to understand the models better than MI.”

**Recommendation 20**

*Future studies should conduct and report sensitivity analyses to test the impact of missing data.*

Twenty respondents considered that this recommendation was “very important”, followed by 13 that considered it “somewhat important.”


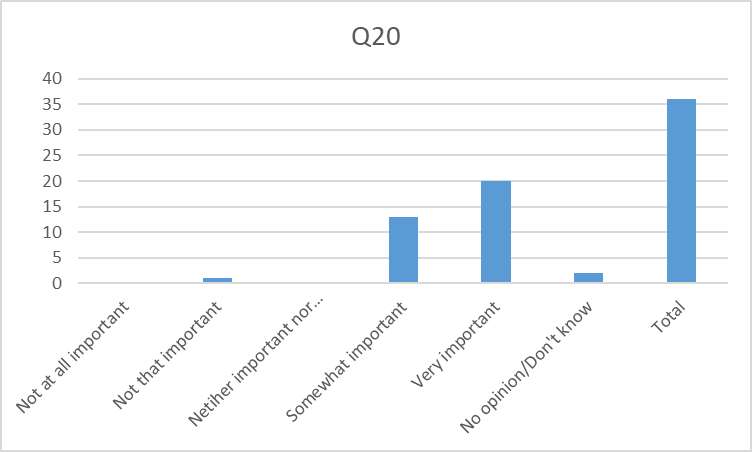


**Open question to recommendation 20**

We had three responses.

“Depends on the amount of missing data, if very small, a sensitivity analysis seems inappropriate”

“Yes, conditional on a cutoff (e.g, if 1% or more of the sample has missing values)”

“This is important and is rarely done.”

**Recommendation 21**

*Future studies should report the proportion of participants lost to follow-up by exposure group.*

Twenty-seven respondents considered that this recommendation was “very important”, followed by nine that considered it “somewhat important.”


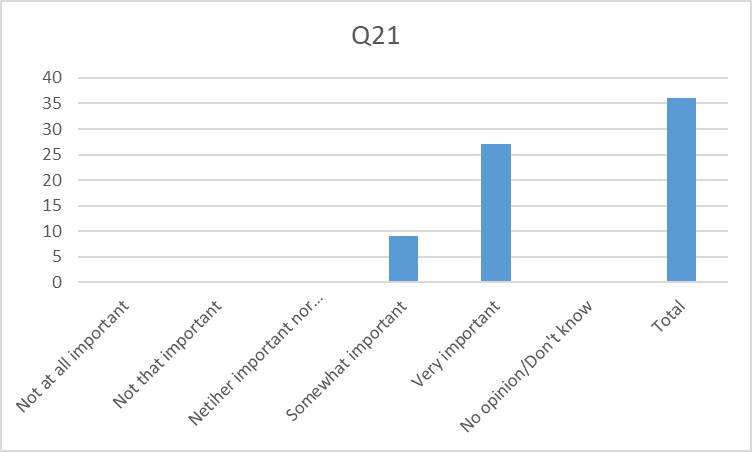


**Open question to recommendation 21**

No responses.

**Recommendation 22**

*Future studies should report the proportion of participants lost to follow-up stratified by characteristics connected to combustible tobacco use (other than the exposure of interest).*

Fifteen respondents considered that this recommendation was “very important”, and fifteen considered it “somewhat important.”


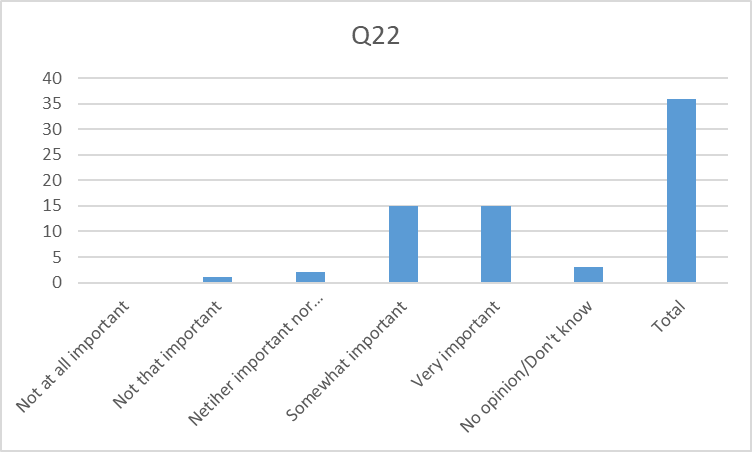


**Open question to recommendation 22**

We had one response.

“I don't quite follow why stratifying by baseline combustible use is important. But I could be persuaded it is; these are subtle data issues.”

## Open question

***“Do you think we’ve missed any important items? If so, please describe here.”***

We had six responses.

“Overall, in the evidence summaries, I hope you will be able to stratify by statistical methods used. i..e Naïve analyses such as regression models vs g-computation or IV methods”

“Future studies should consider type of ENDS products and explore gateways to cigarette smoking or other combustible products”

“No.”

**“**I think it is important that the outcome is regular smoking not smoking initiation, which could literally be one puff on one cigarette ever. Recommendations on a sensible length of follow up (minimum and possibly also maximum) would be useful.”

“There might be an item related to sample construction, in which the researcher explicitly states the inclusion and exclusion criteria used to create the study sample. Another item might relate to sharing data and statistical code.”

“Future research should focus on documenting smoking and vaping control policies the emerging and developing countries.”

## Open question: “Please provide any other overall comments or suggestions you may have.”

We had two responses.

“I know my answers - all "Very Important" - can seem knee-jerkish, but you have provided a clear list of must-haves if the current gap between pro- and anti-harm reduction research is to be breached, at least to some extent.”

“Congrats for doing this! Very important to move the field forward.”

# Process for integrating the feedback from responders into the final recommendations

After the survey was closed, one researcher (MC) conducted the descriptive analysis of results and collated all open-ended comments. These were cross-analyzed against the original list of 22 recommendations. One researcher (MC) created a draft table with all original recommendations, average scores and standard deviations of the Likert scales, and open-ended comments referring to each recommendation. Relevant open-ended comments were signposted with comments (e.g., those that expanded on the content of the draft recommendations such as the ones that resulted in adding the recommendation 23), and a brief summary of the feedback was added to each recommendation (e.g., the notes in table 3 of the manuscript). The draft table was then analyzed with the senior author (JHB) and subsequent tracked edits were made. This version was circulated to the research team, and the final version of the set of 23 recommendations was reached following a few rounds of asynchronous discussion.
